# Elevated Right Hemidiaphragm: A Clue in Acute Cholecystitis?

**DOI:** 10.7759/cureus.14571

**Published:** 2021-04-20

**Authors:** Yucai Yee, Jin Yao Teo

**Affiliations:** 1 Department of Internal Medicine, Singapore General Hospital, Singapore, SGP; 2 Department of Hepato-Pancreato-Biliary and Transplant Surgery, Singapore General Hospital, Singapore, SGP

**Keywords:** complicated acute cholecystitis, chest x ray, diaphragm muscle

## Abstract

Two patients presented to the Emergency Department with sepsis and vague localising complaints. Both of them had a new elevation of the right hemidiaphragm on chest radiography and were eventually diagnosed with complicated acute cholecystitis on CT imaging. In both cases, the hemidiaphragmatic elevation could not be explained by mass effect as there was no sizable intra-abdominal collection. One of the patients was initially misdiagnosed with pneumonia, resulting in clinical deterioration due to delay in definitive management. Awareness of this phenomenon is essential to avoid pitfalls in patients with acute cholecystitis, especially for those who do not present in a typical manner.

## Introduction

In a normal patient, the right hemidiaphragm is within one intercostal space higher than the left on the erect frontal chest radiograph. The new finding of an elevated hemidiaphragm (i.e. when the left hemidiaphragm is higher than the right, or when the right is higher than the left by more than 3cm) [1] is cause for concern and warrants further evaluation. The possible aetiologies are classified by location - whether they are above, below or directly involving the diaphragm [2]. Intra-abdominal causes of raised right hemidiaphragm include infective collections such as hepatic and subphrenic abscesses [3-6], neoplasms such as hepatocellular carcinoma [7,8] or rare tumours such as solitary fibrous tumour [9]. These lesions are typically large and exert a mass effect pushing up the right hemidiaphragm. We discuss the clinical significance of a newly elevated right hemidiaphragm in two patients with acute cholecystitis without mass effect and propose a pathophysiologic mechanism for this finding.

## Case presentation

Case report 1

**Figure 1:**
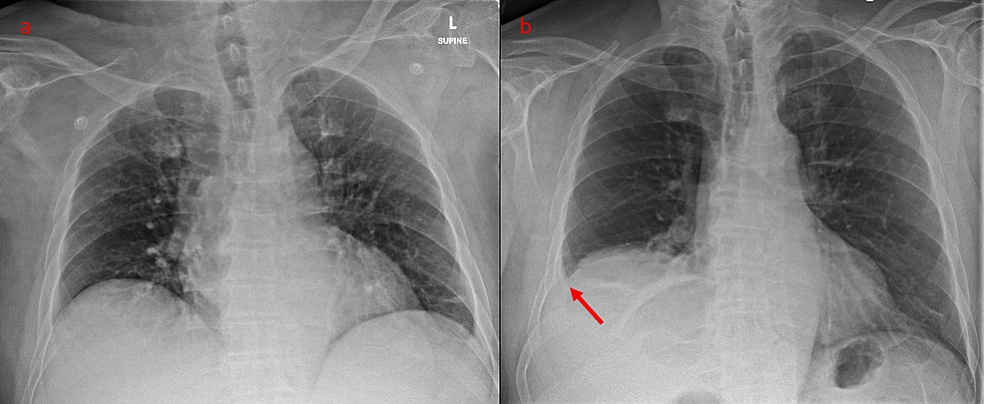
Supine anteroposterior CXR of Case 1 taken three years before admission (a), compared with erect posteroanterior CXR taken on admission (b), showing the development of elevated right hemidiaphragm and small right pleural effusion with basal atelectasis (arrow). CXR - chest X-ray

**Figure 2:**
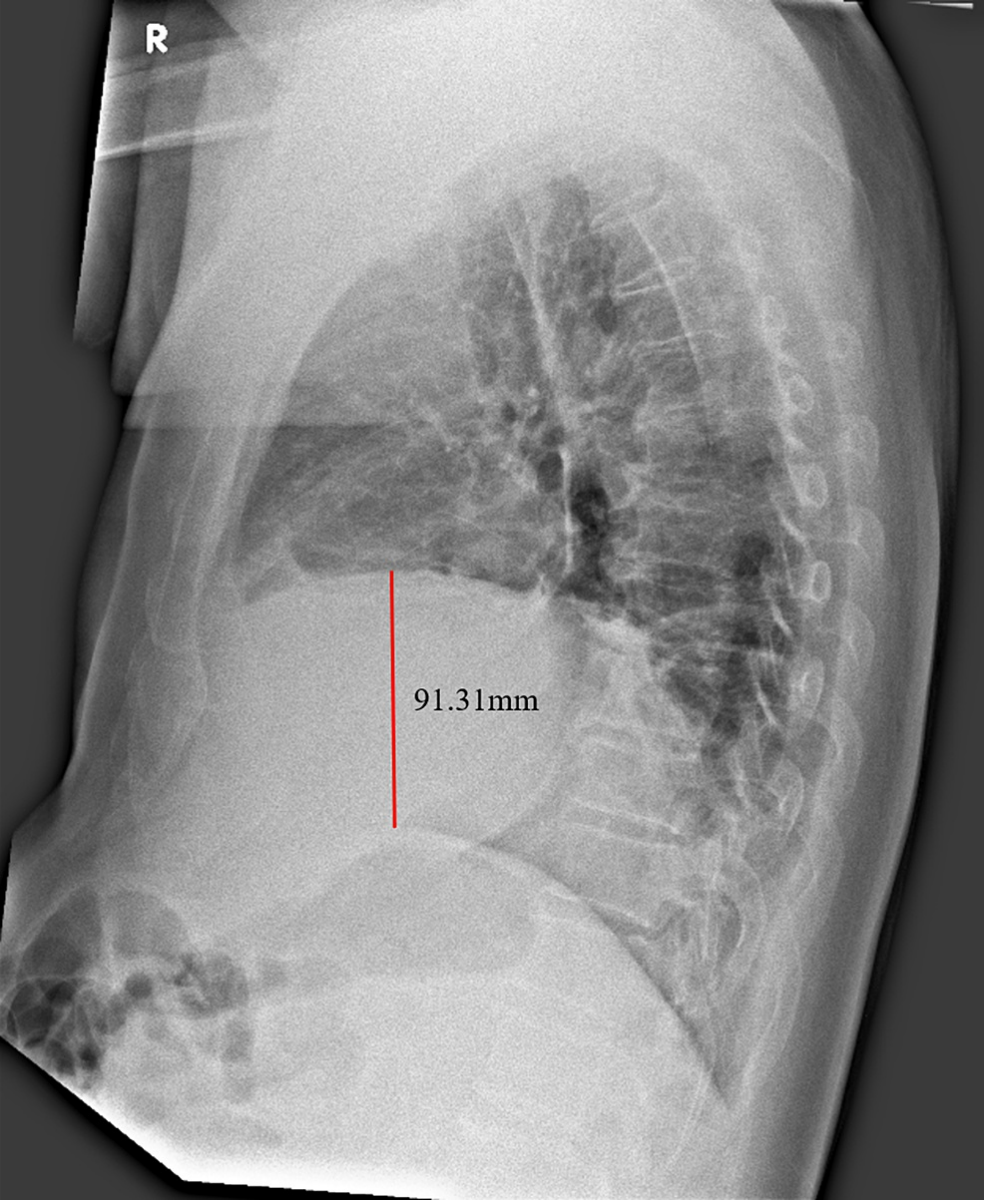
Erect right lateral CXR of Case 1 taken two days after admission. The right hemidiaphragm is markedly higher than the left. A small right pleural effusion is also present. CXR - chest X-ray

Case 1 was a 73-year-old man with a past medical history of hypertension, diabetes mellitus and obesity (BMI 31). He was referred to the Emergency Department (ED) by his general practitioner for concerns of an acute coronary syndrome. In the ED, initial vital signs were temperature 38.8°C, BP 116/64 mmHg, HR 96/min and oxygen saturations 94% on ambient air. He gave a history of epigastric pain for two days, which was non-radiating, worse after food intake and associated with nausea and one episode of vomiting. This was associated with lower right-sided aching chest pain worse on deep inspiration, chesty cough with whitish sputum, blocked nose and fever for the same duration. Physical examination revealed a distended but soft and non-tender abdomen and markedly reduced breath sounds at the right lung base. Initial investigations were significant for low serum sodium of 124mmol/L with serum glucose 13.1mmol/L, elevated neutrophil count of 15.77 x 10^9^/L and elevated inflammatory markers (C-reactive protein 248mg/L, procalcitonin 8.1µg/L). The liver panel, serum amylase and troponin-T with a 12-lead electrocardiogram (ECG) were normal and serum creatinine was at baseline. Erect chest X-ray (CXR) showed a right hemidiaphragm 75mm higher than the left, a new finding compared to a CXR taken three years ago (Figures 1a, 1b). This was confirmed on the erect right lateral CXR (Figure 2).

He was admitted under Internal Medicine with a working diagnosis of right-sided community-acquired pneumonia and gastritis, and started on oral omeprazole, IV ceftriaxone and oral azithromycin after blood cultures were taken. The next day, he presented with persistent high spiking fevers and worsening Type 1 respiratory failure requiring 4L/min intranasal oxygen. A diagnosis of fluid overload was made and he was given a single dose of IV frusemide 40mg. Two hours later, he became hypotensive with BP 91/69, HR 94. Repeat blood tests showed worsening metabolic acidosis and increased serum lactate. Platelet count was downtrending (from 198 x 10^9^/L on admission to 98 x 10^9^/L) but workup for disseminated intravascular coagulation was negative. Frusemide was stopped, and small boluses of IV normal saline were given with response in blood pressure.

**Figure 3:**
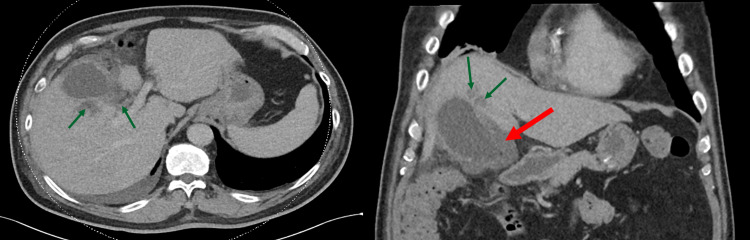
Transverse and coronal CT images of Case 1 showing acute cholecystitis with sealed perforation (red arrow) and adjacent liver abscesses (green arrows). The elevated right hemidiaphragm is also visible on coronal view.

The next morning, a computed tomography (CT) scan of the thorax, abdomen and pelvis was ordered in view of persistent fever and haemodynamic compromise. This revealed a perforated gallbladder with focal areas of loculated fluid collection and intraluminal gallstones, as well as 2-3cm abscesses in adjacent segments four and five of the liver (Figure 3). A small right pleural effusion with atelectasis of the right lower lobe was also present.

The patient was immediately referred to the surgeons, who took over his care. A percutaneous cholecystostomy was performed on day 5 of admission which drained 210mL of brownish fluid over the next two days. His fever subsided after drainage of the biliary fluid. The fluid culture was positive for pansensitive Klebsiella species while multiple blood cultures taken previously were all negative. On day 7, laparoscopic cholecystectomy was performed. Intraoperatively a sealed-off perforation between hepatic flexure to gallbladder with extension medially to the cystic duct and superiorly to gallbladder fossa was noted. The gallbladder was mostly necrotic and friable, with spillage of stones and bile and a large amount of pus evacuated from within. The patient subsequently made an uneventful recovery and was discharged on post-operative day 6.

Case report 2

**Figure 4:**
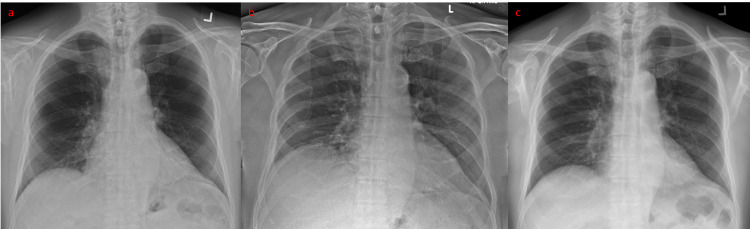
Erect posteroanterior CXR of Case 2 taken one year before admission (a), compared with erect sitting anteroposterior CXR taken on admission (b), showing raised right hemidiaphragm and right basal atelectasis. Repeat CXR taken three months after discharge (c) showed resolution of the raised right hemidiaphragm.

Case 2 was a 77-year-old man with a past medical history of hypertension, diabetes mellitus, hyperlipidaemia, hypothyroidism, intermittent complete heart block (for which he was referred for pacemaker insertion) and nasopharyngeal carcinoma treated with radiotherapy in 2008. He was referred to the ED by the polyclinic as he appeared unwell with slight tachycardia during a routine consult for his chronic medical conditions. In the ED, initial vital signs were temperature 37.6°C, BP 134/84 mmHg, HR 88/min and oxygen saturations 97% on ambient air. Random capillary blood glucose was elevated at 20.2mmol/L. He complained of generalised weakness, loss of weight and appetite for the past two weeks, associated with epigastric pain for three days. There was no diarrhoea, nausea or vomiting. In addition, he had rhinorrhoea for the past few days, on top of a chronic dry cough. Physical examination revealed tenderness over the epigastric and right hypochondrial region without guarding or rebound and reduced air entry over the right lung base. Significant initial investigations included an elevated neutrophil count of 20.34 x 10^9^/L, C-reactive protein 291mg/L, procalcitonin 2µg/L and mildly raised serum creatinine of 121µmol/L (baseline 80-100µmol/L). Erect CXR showed a right hemidiaphragm 60mm higher than the left, not seen on the previous CXR done a year ago (Figures 4a, 4b). Liver panel and serum amylase were normal and 12-lead ECG showed normal sinus rhythm with no conduction block or ischaemic changes. Point-of-care ultrasonography was performed by the ED consultant, who reported a thickened gallbladder wall of at least 8mm with pericholecystic fluid, but no obvious gallstone or sludge.

**Figure 5:**
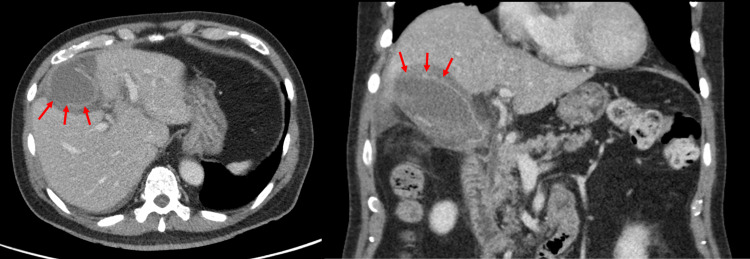
Transverse and coronal CT images of Case 2 showing gallbladder oedema, peri-cholecystic fat stranding and discontinuity of mural enhancement (arrows).

He was started on IV ceftriaxone and metronidazole after blood cultures were taken. In line with the hospital’s COVID-19 policy, he was sent to the acute respiratory infection ward under Internal Medicine to rule out COVID-19 due to the presence of respiratory symptoms and abnormal CXR. A CT scan of the abdomen and pelvis with contrast was ordered on admission, with peri-procedural IV hydration to mitigate the risk of contrast-induced nephropathy. The scan was performed 36 hours later, revealing diffuse gallbladder mural oedema with areas of mural non-enhancement or breach concerning necrosis and small pockets of focal pericholecystic fluid near the fundus (Figure 5). There were ill-defined densities within the gallbladder, which may represent sludge or soft calculi, a moderate amount of pericholecystic fat stranding and a sliver of fluid inferior to the right hepatic edge. There was also a small right pleural effusion with compressive atelectasis in the lower lobe of the right lung.

The patient was referred to the surgeons, who took over his care. He continued to be managed conservatively in view of clinical response to antibiotic therapy, as evidenced by resolution of pain and fever with downtrending neutrophil count. Apart from worsening of glycaemic control due to sepsis requiring the addition of basal insulin during the admission, he made an uneventful recovery and was discharged on day 8 of admission with plans to complete four more days of oral Augmentin after a week of IV antibiotics.

Outcome and Follow-Up

On review in the clinic two weeks post-discharge, Case 1 reported good appetite and his surgical wounds were healing well. Histology of the resected gallbladder showed acute gangrenous cholecystitis with evidence of perforation, without features of dysplasia or malignancy. He was discharged to primary care for continued management of his chronic conditions.

On review in the clinic two weeks post-discharge, Case 2 remained well with no recurrence of abdominal pain or fever, although he had not fully resumed his baseline activities. He was offered a laparoscopic cholecystectomy but declined. An interval CXR taken three months after his discharge for an unrelated condition showed resolution of the raised right hemidiaphragm.

## Discussion

To the best of our knowledge, this is the first report of an association between acute cholecystitis and elevated right hemidiaphragm, without the contribution of mass effect from a concurrent large hepatic or subphrenic collection. We propose that focal peritonitis in acute cholecystitis impairs the normal downward movement of the right hemidiaphragm. This may be more significant in complicated cases, such as those associated with gallbladder gangrene or perforation. The small right pleural effusion and lower lobe atelectasis seen on CT in both cases were likely reactive and not related to a separate pulmonary pathology. On a single frontal CXR, these obscure the dome-shaped contour of the hemidiaphragm, which may result in the clinician not appreciating its raised position. In such a scenario, point-of-care ultrasonography would be useful as the ultrasonographic features of fluid, consolidation and the diaphragm are clearly distinct [10]. The development of respiratory failure in Case 1 was likely contributed by severe sepsis [11] and impairment of lung function due to the elevated right hemidiaphragm, and not pneumonia which he was initially diagnosed with. It is worth mentioning that normal liver function tests do not rule out acute cholecystitis [12] and that held true in these two cases.

## Conclusions

Greater awareness of this phenomenon is crucial to avoid premature closure and diagnostic errors in patients with an atypical presentation of cholecystitis, which can lead to delay in definitive treatment and poor outcomes. Larger scale studies, such as retrospective review of CXRs in confirmed cholecystitis cases, should be performed to elucidate the prevalence of raised right hemidiaphragm as a feature in acute cholecystitis.
